# Engineering stable perovskite X-ray detectors

**DOI:** 10.1038/s41377-022-00969-4

**Published:** 2022-09-14

**Authors:** Kostiantyn Sakhatskyi, Maksym V. Kovalenko

**Affiliations:** 1 Institute of Inorganic Chemistry, Department of Chemistry and Applied Biosciences, ETH Zürich, CH-8093 Zürich, Switzerland; 2 Laboratory for Thin Films and Photovoltaics, Empa – Swiss Federal Laboratories for Materials Science and Technology, CH-8600 Dübendorf, Switzerland

**Keywords:** Photonic devices, Optical materials and structures

*Nature Photonics* **16**, 575–581 (2022) https://www.nature.com/articles/s41566-022-01024-9

The past decade has seen the raise of hybrid organic-inorganic lead halide perovskites as highly potent semiconductors for direct-conversion X-ray detectors. While offering unmatched cost advantages owing to solution growth, and unique defect-tolerant, intrinsic semiconductor characteristics, these materials suffer from reduced operational stability caused by ionic drift and lattice instability. In order to unlock the commercial potential of perovskites, a team of scientists from Sweden and China, led by Prof. Feng Gao (Linköping University, Sweden), Prof. Liang Shen (Jilin University, China), Prof. Kai Yao (Nanchang University, China) turned focus to mixed-cationic perovskites. An extensive chemical engineering and characterization suite pointed to the optimal composition with the combination of cations and doping, reducing the lattice strain and giving raise to lower defect concentrations. For the optimized stoichiometry, single-crystal X-ray detectors exhibited simultaneously high performance, in terms of high sensitivity and low detection limit at low applied voltages, long-term materials stability, and hours of continuous operation. These improvements motivate further investigations into hybrid perovskites, as they no longer appear to substantially lag behind fully inorganic compositions. Moreover, the findings are of high relevance also for other optoelectronic applications of perovskites, including gamma detectors and light-emitting diodes.

